# Effect of Oxygen Tension Modification During Oocyte Maturation on Porcine Oocyte Quality

**DOI:** 10.3390/vetsci12100954

**Published:** 2025-10-03

**Authors:** Yuki Inoue, Saki Akano, Yuya Suzuki, Kota Ushiroshoji, Asuka Kamio, Koumei Shirasuna, Hisataka Iwata

**Affiliations:** 1 Department of Animal Science, Tokyo University of Agriculture, Atsugi City 243-0034, Kanagawa, Japan; yinouye.nodai@gmail.com (Y.I.);; 2 Hanabusa Woman’s Clinic, Kobe City 650-0021, Hyogo, Japan; 3 NODAI Genome Research Center, Tokyo University of Agriculture, Tokyo 156-8502, Japan

**Keywords:** oocyte, oxygen tension, in vitro oocyte maturation, mitochondria, glycolysis

## Abstract

**Simple Summary:**

This study examined the effect of oxygen levels on oocyte metabolism and quality during maturation. The research found that a low-oxygen environment (5% O_2_) throughout maturation did not alter the developmental rate but improved oocyte quality by increasing ATP and lipid content while reducing mitochondrial stress. Gene analysis of cumulus cells revealed that this condition shifted metabolism from oxidative phosphorylation to glycolysis. Furthermore, a two-step oxygen protocol—low oxygen for the first 21 h and high oxygen for the remainder—was shown to be more effective than a consistently high-oxygen environment. This protocol increased ATP and lipid content and enhanced the oocytes’ developmental ability. Ultimately, the study concludes that providing a low-oxygen environment during the initial phase of oocyte maturation is beneficial for improving overall oocyte quality.

**Abstract:**

This study investigated the effects of high (atmospheric) and low (5% O_2_) oxygen tension, as well as a combination of the two, on oocyte metabolism and quality during maturation. Cumulus cell–oocyte complexes collected from gilt ovaries were used for in vitro maturation. In addition, RNA-seq was conducted on the cumulus cells. Low oxygen tension throughout oocyte maturation did not alter the developmental rate to the blastocyst stage; however, it increased oocyte ATP and lipid content while reducing mitochondrial reactive oxygen species and mitochondrial membrane potential. Low-oxygen conditions increased glucose consumption but reduced mitochondrial DNA copy number and mitochondrial protein in cumulus cells. RNA-seq of cumulus cells revealed that low oxygen tension reduced mitochondrial activity and increased glycolysis, with the upregulation of glycolytic genes and downregulation of oxidative phosphorylation and steroidogenesis-related genes. In addition, a two-step oxygen protocol with low (5%) for the first period (0–21 h) and high (20%) for the last half period (21–44 h) increased the ATP and lipid content in oocytes and improved the embryonic developmental ability of the oocytes compared to the high-oxygen group. In conclusion, low oxygen tension during the first part of the maturation period is beneficial for oocyte quality, considering the observed metabolic changes.

## 1. Introduction

Oocytes acquire full developmental competence during growth and final nuclear maturation. In animal embryo production, oocytes from small antral follicles are commonly used as embryo resources. Although the current oocyte maturation system has been established, the quality of in vitro-matured oocytes is still lower than that of in vivo-matured oocytes [1]. In human-assisted reproduction technology, in vivo-matured oocytes are used for embryo production. While the significance of immature oocytes collected during the aspiration of follicles is acknowledged, in vitro maturation conditions are still considered insufficient for high-quality embryo production [2].

Considering the differences between in vivo and in vitro conditions, atmospheric conditions are major candidates for improving oocyte culture conditions. Oocyte maturation occurs in follicles, where the oxygen concentration is half of that in the air [3]. Several studies have investigated the effects of low-oxygen conditions on oocyte maturation. However, contradictory results have been reported: low oxygen tension improves oocyte quality [4,5,6,7], whereas some studies report that it has no effect on oocytes [8,9]. Notably, Whitty et al. showed that the oocyte population in the culture drop and the glucose concentration in the medium are important factors in determining the effect of low oxygen tension, whereas metabolic changes in response to low oxygen tension are considerable factors [10]. Oocytes primarily depend on oxidative phosphorylation using pyruvate; however, the surrounding granulosa cells provide energy substrates via a gap junction [11]. The metabolism of granulosa cells changes in response to hypo-oxygenic conditions by reducing mitochondrial DNA copy number and increasing glucose intake [12]. Bermejo-Alvarez et al. reported that when bovine oocytes are cultured under low oxygen tension, their glucose utilization increases [4]. Based on these reports, modification of oxygen tension during oocyte maturation should take into account the metabolic changes in granulosa cells under low and high oxygen tensions. And the well-balanced combination of the two oxygen tensions would be more suitable for oocyte maturation (two-step culture conditions). Porcine oocyte maturation is an excellent model for studying because it requires a relatively long culture period and can be clearly divided into the germinal vesicle stage and the meiosis stage.

The assisted reproductive technology (ART) of pigs is relatively well established and proposed as a good model for human ART since they share similarities in metabolism and endocrinology [13]. Thus, the aim of this study was to verify the effects of oxygen tension differences on the development and metabolism of porcine oocytes and early embryos, while also examining the underlying genetic variations in CCs. Furthermore, based on these findings, we proposed a novel two-step culture condition for IVM.

In this study, we first examined the effects of high and low oxygen tension throughout the maturation period on porcine oocyte quality and the metabolism of cumulus cells (CCs) and oocytes. Second, we modified the current culture conditions and found that a two-step culture condition with high oxygen tension for the first period and low tension for the last period was useful for in vitro oocyte maturation.

## 2. Materials and Methods

### 2.1. Chemicals and Media

Unless otherwise stated, all chemicals used in this study were purchased from Nacalai Tesque (Kyoto, Japan). All media and solutions were prepared in the laboratory according to previously described protocols, unless stated otherwise.

### 2.2. Cumulus Cell–Oocyte Complex (COC) Collection and In Vitro Maturation

This study was designed in accordance with the ARRIVE guidelines and approved by the Ethics Committee for Animal Experiments of Tokyo University of Agriculture (approval number: 2023009).

**Figure 1:**
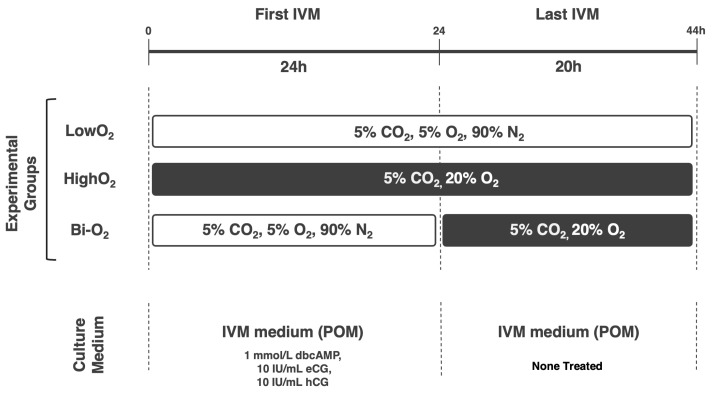
Atmospheric condition used in each experiment.

Porcine ovaries were collected from gilts (LWD, about 180 days age, 100–120 kg) and transported to the laboratory within 1 h in phosphate-buffered saline (PBS) at 38 °C. Collection of COCs and oocyte maturation were performed as previously described [14]. Oocytes collected from the slaughterhouse-derived ovaries were pooled (at least 500 COCs) and randomly allocated to each experimental group. In every replicate, fifty pairs of ovaries were collected at the slaughterhouse. The basal medium for in vitro maturation (IVM) of oocytes was the porcine oocyte medium (POM) supplemented with 10% *v*/*v* porcine FF, 0.5 mmol/L l-cysteine, 10 ng/mL epidermal growth factor (Sigma-Aldrich, St Louis, MO, USA), and 3 mg/mL polyvinyl alcohol (PVA) [15]. Porcine FF was aspirated from the antrum follicles (3–5 mm in diameter) of gilts, centrifuged at 10,000× *g* for 5 min, filtered with 0.22 μm, and stored at −20 °C until use. As described in previous reports [14], the IVM process was divided into two steps: in the first IVM step, the COCs were cultured in the IVM medium containing 1 mmol/L Dibutyryl-cAMP (dbcAMP), 10 IU/mL equine chorionic gonadotropin (eCG; ASKA Pharma Co., Ltd., Tokyo, Japan), and 10 IU/mL human chorionic gonadotropin (hCG; Fuji Pharma Co., Ltd., Tokyo, Japan) for 24 h, and in the last IVM step, the COCs were cultured without dbcAMP and hormones for 20 h. The IVM was performed at 38.5 °C under differential atmospheric conditions. The oxygen conditions used in this study are summarized in Figure 1; In the first experiment, high oxygen tension (HighO_2_; 5% CO_2_ in air) or low oxygen tension (LowO_2_; 5% CO_2_, 5% O_2_, and 90% N2) was used. In the second experiment, we used HighO_2_ conditions and 2-step O_2_ conditions: Bi-O_2_; LowO_2_ conditions for the first half of IVM and HighO_2_ conditions for the last half of IVM.

### 2.3. Parthenogenetic Activation (PA) of Oocytes and In Vitro Culture (IVC) of Embryos

Polyspermy frequently occurs in porcine oocytes, which can compromise data accuracy. In this study, to avoid this issue, we used PA to evaluate embryonic development. After IVM, oocytes were denuded of cumulus cells by vortexing with 0.1% (*w*/*v*) hyaluronidase and subjected to PA. Oocyte activation was performed by a single electrical pulse of 60 V for 0.1 ms using the CUY500P1 electrode (NEPA21; NepaGene Ltd., Chiba, Japan) in 280 mmol/L mannitol containing 0.05 mmol/L CaCl_2_ and 0.1 mmol/L MgSO_4_. Activated oocytes were incubated in porcine zygote medium 3 (PZM-3) supplemented with 10 μmol/L cytochalasin B and 10 μmol/L cycloheximide for 4.5 h and then transferred to PZM-3 for culture (10 embryos in each 10-μL drop) [16]. Embryos were cultured at 38.5 °C under low oxygen tension.

### 2.4. Evaluation of Metaphase II (MII) Oocyte Maturation Rate, Cleavage Rate, and Blastulation Rate

After IVM, the MII rate was determined by the presence of a polar body and clear spindle, confirmed by 10 μg/mL Hoechst 33342 staining. The cleavage rate was evaluated two days after PA, and the blastulation rate was evaluated seven days after PA. The total cell numbers in the blastocysts were determined by Hoechst 33342 staining.

### 2.5. Evaluation of ATP Content in the Oocytes and Blastocyst

ATP content in individual oocytes was measured using ATP-dependent luciferin-luciferase bioluminescence assay (ATP assay kit; Toyo-Inc., Tokyo, Japan), as described previously [17].

### 2.6. Evaluation of Glucose Consumption in Bi-Phasic IVM

At the end of both the first and last IVM periods, the culture medium was collected, and glucose concentration was measured using LabAssay (TM) glucose (FUJIFILM Wako, Osaka, Japan). Glucose consumption was calculated by subtracting the glucose concentration in the post-incubation medium from that in the basal medium.

### 2.7. Lipid Content in the MII Oocytes and Blastocysts

Lipid content in MII oocytes was determined using Nile Red (Wako, Osaka, Japan) and Hoechst 33342 as described previously [16]. The samples were mounted on glass slides with an antifade medium (Immunoselect Antifading Mounting Medium; Dianova, Hamburg, Germany) and observed under a fluorescence microscope (Leica DMI 6000 B microscope using LAS AF software Version1.2; Leica, Wetzlar, Germany). Digital images of the equatorial regions of the samples were analyzed using ImageJ software (ver.2.14.0, NIH, Bethesda, MD, USA).

### 2.8. Measurement of the Mitochondrial DNA Copy Number (Mt-cn)

Mt-cn expression in individual oocytes or cumulus cells was determined by real-time quantitative PCR (RT-qPCR), as described previously [14]. Briefly, cumulus cells were isolated from 50 COCs using 0.1% (*w*/*v*) hyaluronidase and centrifuged to obtain cellular pellets. DNA was extracted from individual oocytes and cells. A primer set targeting the mitochondrial genome was designed using Primer 3Plus (NCBI reference: NC_000845.1; forward: ATCCAAGCACTATCCATCACCA; reverse: CCGATGATTACGTGCAACCC). PCR was performed using a real-time PCR system (Bio-Rad Laboratories, Hercules, CA, USA) and the KAPA SYBR Fast qPCR Master Mix (Kapa Biosystems, Wilmington, MA, USA). The thermal program consisted of the following steps: 95 °C for 3 min, followed by 40 cycles of 98 °C for 5 s, and 60 °C for 11 s. Serially diluted vectors containing the corresponding PCR products were used as standards, and the vector was sequenced by Sanger sequencing before use. The Mt-cn of cumulus cells was normalized using cumulus cell numbers determined by PCR targeting 1-copy genes (targeting beta-actin(ACTB); forward: AATGGGAACGTGGGTAGCAG, reverse: AGGCTCAGAGGATCAGCTGA).

### 2.9. Evaluation of the Mitochondrial Membrane Potential (MMP)

The oocytes were incubated in a culture medium containing 10 μmol/L of JC-1 dye (Invitrogen) for 30 min at 38.5 °C under each oxygen condition. The samples were mounted on glass slides with an antifade medium and observed under a fluorescence microscope (Leica DMI 6000 B microscope using LAS AF software). Digital images of all mitochondria (green fluorescence) and active mitochondria (red fluorescence) were captured, and fluorescence intensity was analyzed using ImageJ software. The red/green ratio was calculated for the individual samples.

### 2.10. Immunoblot Analysis

Cumulus cells collected from 100 COCs were used to analyze protein expression using Western blotting (WB). The cumulus cells were lysed in 100 μL lysis buffer containing Complete Tablets Mini EASYpack and PhosSTOP EASYpack (Roche, Basel, Switzerland), homogenized, removed from the debris, and centrifuged at 20,400× *g* for 30 min after homogenizing. The total protein concentration of each sample was measured using a Pierce BCA Protein Assay Kit (Thermo Fisher Scientific, Waltham, MA, USA) and diluted by distilled water. The samples diluted in 2x Laemmli sample buffer (Bio-Rad) containing 2-Mercaptoethanol (20 ng per each lane) followed by heating at 95 °C for 5 min. The samples were resolved on 15% SDS-PAGE gels and transferred onto PolyVinylidene DiFluoride (PVDF) membranes. The membranes were rinsed using TBS-T and blocked using Block Ace (KAC Co., Ltd., Kyoto, Japan) at 37 °C for 1 h. Then, the membranes were incubated with primary antibodies overnight at 4 °C, followed by secondary antibodies for 1 h at room temperature. The list of antibodies and their dilution rates is shown in Supplementary Table S1. Each antibody was diluted using Signal Booster Solutions A and B (Beacle Inc., Kyoto, Japan). Antibodies were detected using Western ECL substrate (Bio-Rad) and observed using ImageQuant LAS 4000 (GE Healthcare, Chicago, IL, USA). After the measurements, the membranes were stripped of all antibodies using a stripping solution (FUJIFILM Wako) and used for protein analyses. The protein expression levels of TOMM20 were normalized to the expression level of ACTB, and the expression of mitochondrial proteins was normalized to the expression level of translocase of outer mitochondrial membrane 20 (TOMM20).

### 2.11. RNA-Sequence (RNA-Seq) of Cumulus Cells from IVM

After IVM, cumulus cells from 100 COCs were subjected to RNA-Seq. Three samples were prepared from each experimental group using differential ovary series. RNA was extracted using RNAqueous™-Micro (Thermo Fisher) and the quality and concentration of total RNA was determined using a 2100 Bioanalyzer (Agilent Technologies, Santa Clara, CA, USA). The average RNA quality index was 9.45 ± 0.13. Library preparation was performed using the NEBNext Ultra II Directional RNA Library Prep Kit (New England Biolabs, Ipswich, MA, USA). The concentrations of the cDNA libraries were determined using an Agilent high-sensitivity DNA kit and a Bioanalyzer 2100 (Agilent Technology, Santa Clara, CA, USA). The concentrations of the cDNA libraries were reassessed using a Kapa Library Quantification Kit (Kapa Biosystems, Wilmington, MA, USA). The multiplexed samples were sequenced in single-read 100-bp reads using the NextSeq1000 system (Illumina, San Diego, USA). Raw data were generated using the bcl2fastq2 software v2.18.0.12 (Illumina) according to the manufacturer’s instructions. Raw sequence processing, reference genome mapping, and differential gene expression analysis were performed using the CLC Genomics Workbench ver. 23.0.2 (Qiagen, Hilden, Germany). The sequence data were aligned to the Sus scrofa genome sequence (Sscrofa11.1: GCF_000003025.6) to count the sequence reads. Gene expression values were expressed as Transcripts Per Million (TPM) > 0. Differentially expressed genes (DEGs) were determined using transcriptomic tools from the CLC Genomics Workbench with a threshold False Discovery Rate (FDR) Q value of <0.05. Kyoto Encyclopedia of Genes and Genomes (KEGG) pathway enrichment analysis of DEGs were performed using the DAVID functional annotation tool (https://davidbioinformatics.nih.gov/tools.jsp (accessed on 10 September 2025)), with *Sus scrofa* as the background species. The fold enrichment score was calculated using the geometric mean of the log scale of the member’s *p* value in the corresponding annotation. *p*-values were calculated using Fisher’s exact test. The data have been deposited in the DNA Data Bank of Japan (DDBJ) BioProject database under BioProject accession number PRJDB35842.

### 2.12. Statistical Analysis

All data sets were subjected to data analysis. For data analyses, data distribution was examined using the Shapiro–Wilk test. For data with a normal distribution, the Welch–Aspin *t*-test with two tails was used to determine the statistical significance of differences between the two groups. Samples deviating from the normal distribution following the normality test were removed using Grubbs’ test. Non-parametric data were analyzed using the Mann–Whitney U test. Statistical significance was set at *p* < 0.05.

## 3. Results

### 3.1. Developmental Ability and Mitochondrial Function in Oocytes

Oocyte maturation, cleavage, or the developmental rate up to the blastocyst stage was not affected by LowO_2_ conditions. In addition, the total number of blastocysts was comparable between the two groups (Table 1).

**Figure 2:**
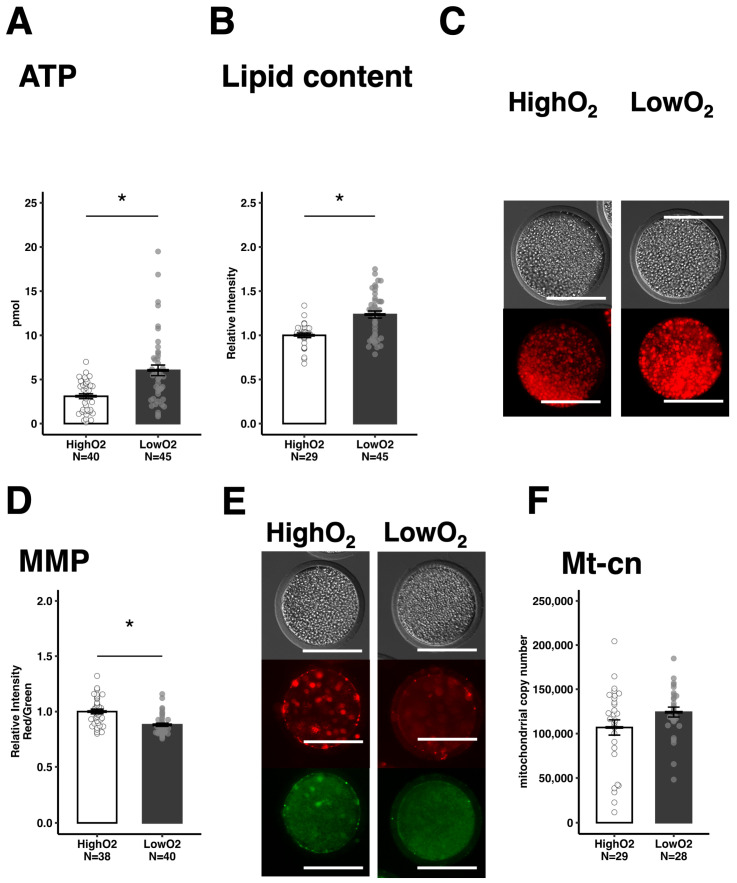
Effect of differential oxygen tensions (HighO_2_ or LowO_2_) on ATP and lipid content, and mitochondria in oocytes. ATP content in the metaphase II (MII) stage oocytes (**A**). Relative lipid contents in the MII stage oocytes (**B**) and representative images (**C**). Mitochondrial membrane potential (MMP) in the MII stage oocytes (**D**) and representative images (**E**). The fluorescence intensity of MMP was measured using the JC-1 red (active mitochondria)/green (all mitochondria) ratio. Mitochondrial DNA copy numbers (Mt-cn) in the MII stage oocytes (**F**). Data are presented as the mean ± standard error of the mean, and the value of HighO_2_ groups were defined as 1.0 in (**B**,**D**); * *p* < 0.05. Scale bar indicates 100 μm.

After the IVM period, both ATP and lipid contents in oocytes were higher under the LowO_2_ condition than under the HighO_2_ condition (Figure 2A–C). The high-energy status of oocytes prompted us to examine the mitochondrial activity. The LowO_2_ condition decreased MMP (Figure 2D,E), whereas Mt-cn levels were comparable between the groups. (Figure 2F).

### 3.2. Metabolism of Cumulus Cells Under Two Oxygen Conditions

**Figure 3:**
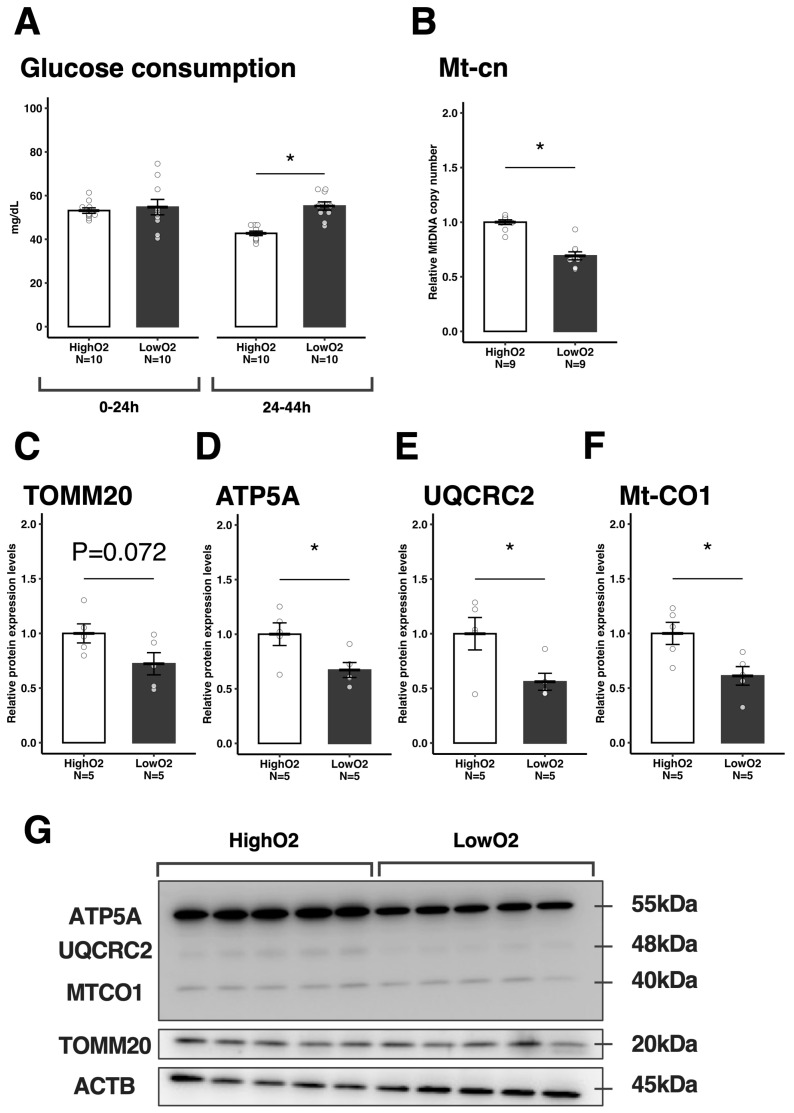
The effect of differential oxygen tensions on cumulus cell metabolism and mitochondrial parameters. The glucose consumption of COCs determined at the end of the first (0–24 h) or the last (24–44 h) maturation period (**A**). The Mt-cn in the cumulus cells (**B**). Relative protein expression levels of TOMM20 (**C**), ATP5A (**D**), UQCRC2 (**E**), and MTCO1 (**F**) in the cumulus cells, and representative membrane images (**G**). Except for Mt-cn, the value of the control group was defined as 1.0. The data are presented as mean ± standard error of the mean; * *p* < 0.05.

Since there was no difference in mitochondrial number and low mitochondrial activity in oocytes, but there was greater ATP and lipid content in oocytes under the LowO_2_ condition, we explored the effect of oxygen tension on cumulus cells. First, we found that glucose consumption in the medium was comparable in the first IVM period but significantly higher in the LowO_2_ condition during the last IVM period (Figure 3A). Furthermore, Mt-cn in cumulus cells was significantly less under the LowO_2_ condition (Figure 3B), and the relative protein expression levels of TOMM20 tended to be low under the LowO_2_ condition (Figure 3C,G). Moreover, the relative expression levels of each mitochondrial protein [ATP5A (Complex V), UQCRC2 (Complex IV), and MTCO1 (Complex III)] were lower in the LowO_2_ condition (Figure 3D–G) compared to their HighO_2_ counterparts.

**Figure 4:**
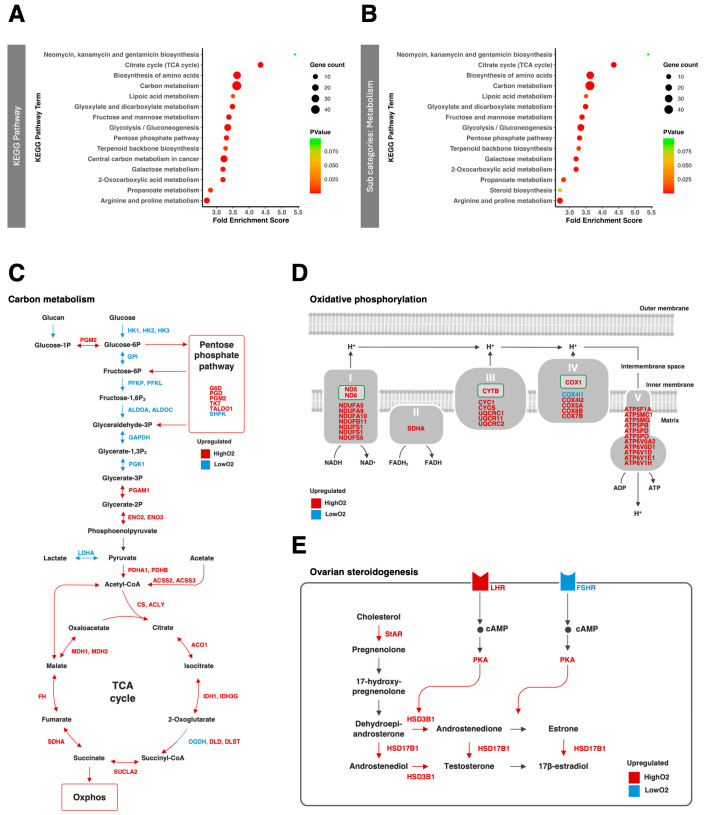
The RNA-seq of the cumulus cells. The top 15 KEGG pathways analysis (**A**) and subcategories of metabolism (**B**). The pathways were enriched by the differentially expressed genes with fold enrichment score (color scale: fold enrichment score; dot size: gene counts). (**C**–**E**) show expression of genes (blue: upregulated by LowO_2_ condition; red: downregulated by LowO_2_ condition) regarding carbon metabolism (**C**), oxidative phosphorylation (**D**) and ovarian steroidogenesis (**E**).

Next, we conducted RNA-seq using cumulus cells derived from COCs after IVM under high or low O_2_ conditions. RNA-seq revealed that 2173 genes (1099 upregulated under LowO_2_ conditions and 1074 upregulated under HighO_2_ conditions) were differentially expressed between the two oxygen conditions. Gene Ontology (GO) analysis showed some unique GO terms, which included carbon metabolism (glycolytic process, tricarboxylic acid cycle, and carbohydrate metabolic process), reactive oxygen species (ROS) metabolic process, and response to oxidative stress (Figure S1A). Similarly, the KEGG pathway analysis showed that carbon metabolism (citrate cycle, carbon metabolism, glyoxylate and dicarboxylate metabolism, fructose and mannose metabolism, glycolysis/gluconeogenesis, pentose phosphate pathway, central carbon metabolism in cancer, and 2-Oxocarboxylic acid metabolism) was enriched in the DEGs (Figure 4A). Among the metabolic subcategories of the KEGG pathway, steroid biosynthesis was newly enriched as a specific pathway related to differential oxygen tension (Figure 4B). The other subcategories are shown in Figure S1B.

DEGs related to carbon metabolism and oxidative phosphorylation are shown in Figure 4C,D. Genes associated with glycolysis were upregulated under the LowO_2_ condition whereas genes associated with the TCA cycle, pentose phosphate pathway, and oxidative phosphorylation (OXPHOS) were downregulated under the LowO_2_ condition. Moreover, steroidogenesis within follicle cells in response to follicle stimulating hormone (FSH) and luteinizing hormone (LH) is the main driver of antral follicle growth and oocyte meiosis [18]. Only the follicle-stimulating hormone receptor (FSHR) was upregulated by theLowO_2_ condition, and most of the genes related to steroid synthesis and luteinizing hormone receptor (LHR) were downregulated by the LowO_2_ condition (Figure 4E).

### 3.3. Novel Two-Step Culture Oxygen Condition Improves Oocyte Quality

**Figure 5:**
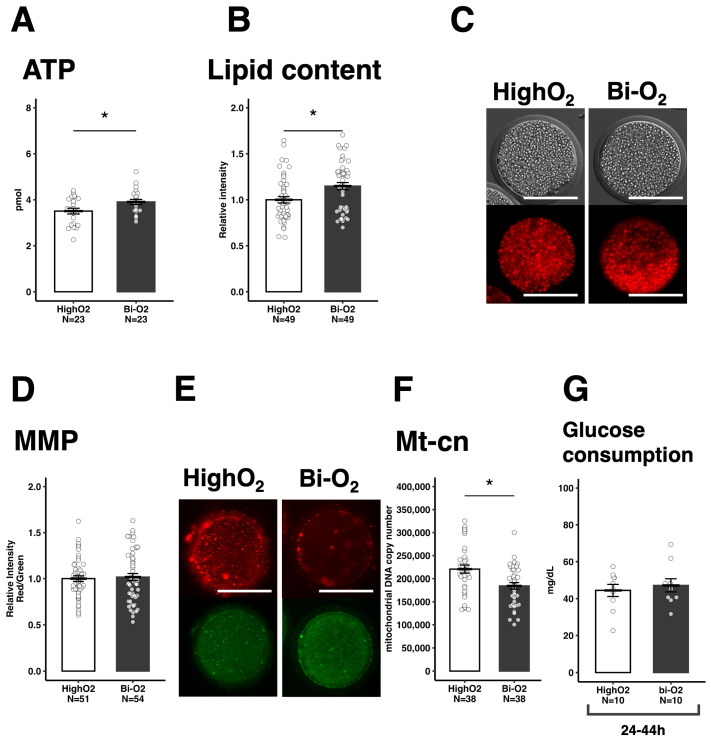
The effect of the two-step oxygen condition (Bi-O_2_ condition) on the energy state and mitochondria in oocytes. ATP content in the metaphase II (MII)-stage oocytes (**A**). Relative lipid contents in the MII stage oocytes (**B**) and representative images (**C**). The mitochondrial membrane potential (MMP) in the MII stage oocytes (**D**) and representative images (**E**). The fluorescence intensity of MMP was measured using the JC-1 red (active mitochondria)/green (all mitochondria) ratio. The mitochondrial DNA copy numbers (Mt-cn) in the MII stage oocytes (**F**). The glucose consumption in the medium after IVM (**G**). The data are presented as the mean ± standard error of the mean; * *p* < 0.05. Scale bar indicates 100 μm.

Based on the above-described results, a hypothesis was raised: LowO_2_ conditions were beneficial for high glycolytic ability but suppressed mitochondrial activity and ovarian steroid hormone production in CCs. A recent study has indicated that high levels of ATP supplied by CC mitochondria in response to LH are important for oocyte competence [19]. Therefore, biphasic oxygen conditions are optimal for oocyte maturation, where the oxygen tension changes from low to high at certain time points during maturation (Bi-O_2_, details of which are shown in Figure 1). The Bi-O_2_ condition tended to improve oocyte maturation (*p* = 0.092) and significantly improved the blastocyst stage than the HighO_2_ condition (Table 2). ATP and lipid contents in oocytes were also higher in the Bi-O_2_ condition than in the HighO_2_ condition (Figure 5A–C). Although MMP was comparable between the two culture conditions (Figure 5D,E), Bi-O2 decreased Mt-cn (Figure 5F). Moreover, the glucose consumption of COCs was comparable between the Bi-O_2_ and HighO_2_ conditions at the end of the IVM period (Figure 5G).

## 4. Discussion

The present study shows that low-oxygen conditions change mitochondrial function in oocytes and increase glucose usage in cumulus cells, which in turn increases ATP and lipid content in oocytes. Considering these changes in oocytes and cumulus cells, a novel two-step oxygen condition for oocyte maturation was shown to significantly improve the developmental ability of oocytes with a higher energy status compared to conventional maturation conditions (high tension).

Our results showed that lowering the oxygen concentration increased the ATP content in oocytes. Oocytes depend on oxidative phosphorylation for energy production; however, the present study showed that low O_2_ tension reduced mitochondrial activity with no changes in mitochondrial DNA content in oocytes. These results indicate that the high ATP content in oocytes is caused by the surrounding cumulus cells because these cells provide energy via gap junctions [20,21].

Kansaku reported that Carbonyl cyanide m-chlorophenyl hydrazone (CCCP; mitochondrial membrane uncoupler)-treated denuded bovine oocytes reduced ATP content in oocytes, but did not affect that in granulosa cells, suggesting that cumulus and granulosa cells mainly use glycolysis for energy production [22]. Furthermore, lowering the oxygen concentration increases glucose use in bovine granulosa cells [12]. Consistently, in our experiment, glucose usage by COCs increased under low-oxygen conditions. Furthermore, RNA-seq showed that the LowO_2_ condition upregulated genes associated with glycolysis pathways and downregulated genes associated with the pentose phosphate pathway and the TCA cycle. Therefore, low-oxygen conditions increase the glycolytic activity in cumulus cells.

The lipid content in oocytes is reported to be closely related to the number of cumulus cells surrounding the oocytes [23]. Lipids in oocytes increase during oocyte growth and removing them from oocytes reduces their developmental ability [24,25]. These reports and the present results suggest that a high lipid content under low-oxygen conditions reflects high oocyte quality.

In many trials using low-oxygen conditions for oocyte maturation, no consistent results have been reported [5,10,26]. This may be due to a lack of understanding of the metabolism of cumulus and granulosa cells under differential oxygen conditions and its relationship with oocyte quality.

Morimoto et al. reported that the metabolism of cumulus and granulosa cells changes during oocyte maturation such that glycolysis and mitochondrial respiration sharply increase after hCG treatment in mice [19]. Interestingly, this report also showed that in the final stage of oocyte maturation, the ratio of mitochondrial ATP production in granulosa cells is positively related to embryo production outcome, and the ratio of glycolytic ATP production is inversely related to embryonic production [19]. Our RNA-seq analysis revealed that low-oxygen conditions increased glycolysis and decreased the expression of genes associated with mitochondria, which play an important role in steroidogenesis and oxidative phosphorylation. In addition, the number of capillary blood vessels extending to the antral follicles drastically induces follicle development following eCG treatment [27]. These reports indicate that high-oxygen conditions that support OXPHOS are important from a certain point in the oocyte maturation period.

Considering these results, we modified the culture method to a two-step maturation condition, in which the atmospheric conditions were under low oxygen tension in the first half of the maturation period and were under high oxygen tension in the last half of maturation period. This modification yielded a significantly higher rate of blastocyst formation and oocyte quality. These oocytes had high ATP and lipid content, indicating that low oxygen tension until a certain time point may be beneficial for increasing the energy status of oocytes. In contrast, the MMP in oocytes and glucose consumption by COCs were comparable between the groups. The mitochondrial DNA content in oocytes was still low in the Bi-O_2_ group compared to that in the HighO_2_ group, suggesting that there may be optimal time points for changing oxygen tension during IVM.

The oxygen tension in the follicles or oviduct is significantly lower than that of atmospheric oxygen or blood [28,29,30,31]. This characteristic environment in the follicles can induce angiogenesis, mediated by vascular endothelial growth factor (VEGF), which facilitates the supply of essential nutrients, oxygen, and steroids necessary for further follicular growth [32]. Previous studies have reported that culturing isolated granulosa cells (GCs) under hypoxic conditions promotes metabolism and cell proliferation [33]. Consistently, our RNA-seq data support these findings, demonstrating an increase in VEGF expression (fold change, 4.24; FDR Q-value, 2.51 × 10^−108^) and corresponding changes in related pathways under low-oxygen conditions.

Unfortunately, measuring oxygen tension within follicles during the transition from the antral to the Graafian follicle stage is difficult, and there are no reports regarding the kinetics of follicular oxygen tension in vivo. Redding et al. predicted that oxygen tension increases during the ovulatory phase in human follicles based on mathematical modeling [34]. Here, we show that higher oxygen tension at the final maturation stage is beneficial; however, it is unclear whether the two-step oxygen culture condition mimics the in vivo environment. This point remains to be addressed in future studies.

## 5. Conclusions

To the best of our knowledge, this is the first report showing that modification of atmospheric conditions in a two-step manner supports oocyte maturation. This knowledge is useful for establishing optimal maturation conditions under which oxygen tension is modified according to the demand for oxidative phosphorylation and glycolysis in COCs and may contribute to improving IVM protocols.

## Figures and Tables

**Table 1:** Effect of differential oxygen tension on maturation and developmental competence.

Groups	No. of Oocytes	No. of Trials	MII (%)	No of Oocytes	No. of Trials	4 Cell (%)	Blastocysts (%)	Cell No. (No.)
HighO_2_	141	3	82.2 ± 2.8	176	17	58.0 ± 3.7	15.6 ± 1.4	38.6 ± 3.8 (23)
LowO_2_	146	3	80.0 ± 4.5	183	17	63.6 ± 3.2	19.2 ± 2.1	39.5 ± 2.9 (26)

**Table 2:** Effect of bi-oxygen phase on maturation and developmental competence.

Group	No. of Oocytes	No. of Trials	MII (%)	No. of Oocytes	No. of Trials	4 Cell Rate (%)	Blastocyst Rate (%)		Cell No. (No.)
HighO_2_	141	3	82.2 ± 2.8	320	32	43.8 ± 3.5	15.6 ± 2.5	a	55.3 ± 3.6 (39)
Bi-O_2_	153	3	91.6 ± 3.2	320	32	49.7 ± 3.3	24.1 ± 2.3	b	53.4 ± 2.4 (50)

## Data Availability

The data presented in this study are openly available in DNA Data Bank of Japan with links to the BioProject at https://www.ddbj.nig.ac.jp/index-e.html (accessed on 10 September 2025), reference number PRJDB35842.

## Supplementary Materials

The following supporting information can be downloaded at https://www.mdpi.com/article/10.3390/vetsci12100954/s1, Figure S1: The RNA-seq of the cumulus cells. The top 15 GO terms for biological processes predicted by GO analysis (A). GO terms were enriched in differentially expressed genes according to the fold enrichment score (color scale: fold enrichment score; dot size: gene counts). The top 15 KEGG pathway analyses (B) and subcategories of metabolism and environmental information processing (C). The pathways were enriched by differentially expressed genes with fold enrichment scores (color scale, fold enrichment score; dot size, gene counts). Each gene (blue: upregulated by LowO_2_ condition; red: downregulated by LowO_2_ condition) was associated with carbon metabolism (D), oxidative phosphorylation (E), and ovarian steroidogenesis (F). Figure S2: Original image of mitochondrial protein (ATP5A, UQCRC2 and MTCO1) using Total OXPHOS Rodent WB Antibody Cocktail. Figure S3: Original image of TOMM20. Figure S4: Original image of ACTB. Table S1: The list of antibodies.

## Abbreviations

ACTB	Beta actin
ATP5A	ATP synthase lipid-binding protein V
Bi-O_2_	2-step O_2_ condition: Bi-O_2_
CCs	Cumulus cells
CCCP	Carbonyl cyanide m-chlorophenyl hydrazone
COCs	Cumulus cell-oocyte complexes
dbcAMP	Dibutyryl-cAMP
DEGs	Differentially expressed genes
eCG	Equine chorionic gonadotropin
FDR	False Discovery Rate
FSH	Follicle stimulating hormone
FSHR	Follicle stimulating hormone receptor
GCs	Granulosa cells
hCG	Human chorionic gonadotropin
HighO_2_	High oxygen tension
IVC	In vitro culture
IVM	In vitro maturation
KEGG	Kyoto Encyclopedia of Genes and Genomes
LH	Luteinizing hormone
LHR	Luteinizing hormone receptor
LowO_2_	Low oxygen tension (LowO_2_
MII	Metaphase II
MMP	Mitochondrial membrane potential
Mt-cn	Mitochondrial DNA copy number
MT-CO1	Cytochrome c oxidase subunit I
PA	Parthenogenetic activation
PBS	Phosphate-buffered saline
POM	Porcine oocyte medium
PVA	Polyvinyl alcohol
PZM-3	Porcine zygote medium 3
ROS	Reactive oxygen species
TCA	Tricarboxylic Acid
TOMM20	Translocase of outer mitochondrial membrane 20
TPM	Transcripts Per Million
UQCRC2	Ubiquinol-cytochrome c reductase core protein II
VEGF	vascular endothelial growth factor
WB	Western blotting
