# Supplementary material for: Effect of Oxygen Tension Modification During Oocyte Maturation on Porcine Oocyte Quality
Source: Vet Sci. 2025 Oct 3;12(10):954. doi: 10.3390/vetsci12100954 (PMC12568154; doi:10.3390/vetsci12100954)
Supplement: Supplementary file 1 [file vetsci-12-00954-s001.zip › vetsci-3896498-supplementary (1).pdf]

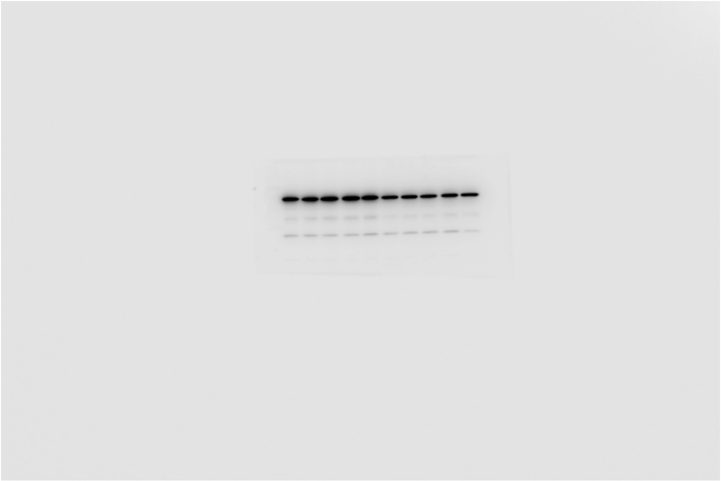

Figure S2

Original image of mitochondrial protein(ATP5A, UQCRC2 and MTCO1) using Total OXPHOS Rodent WB Antibody Cocktail.

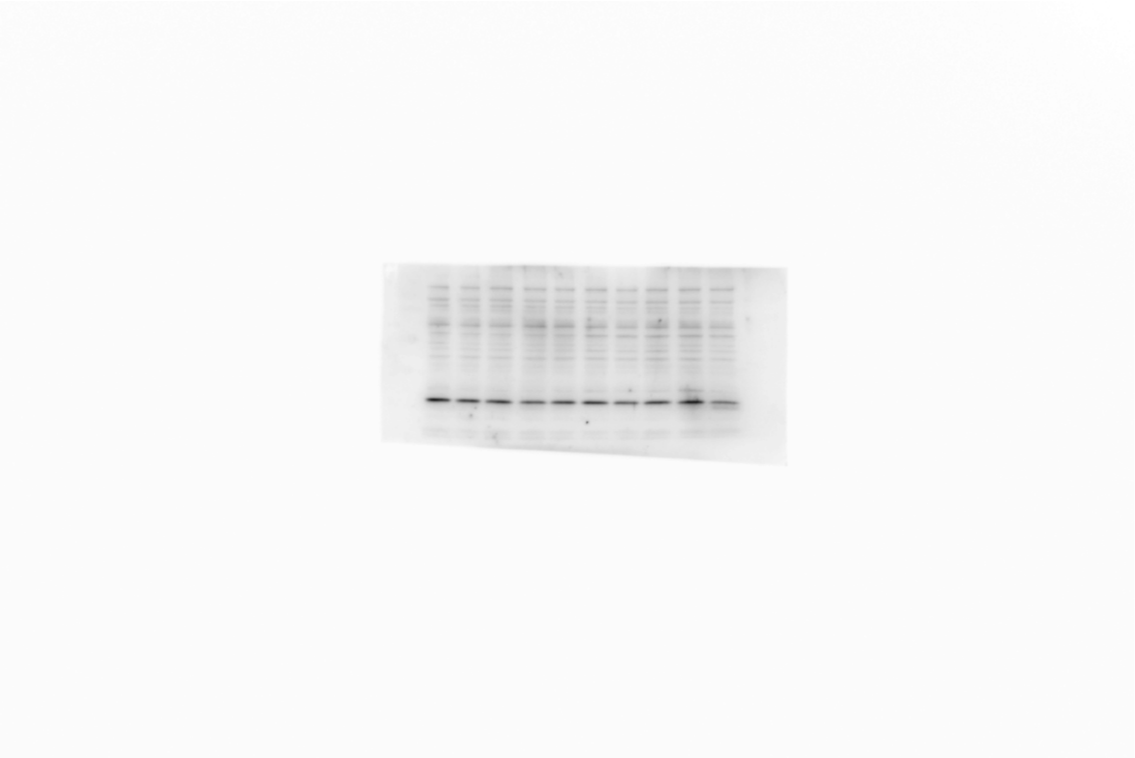

Figure S3

Original image of TOMM20.

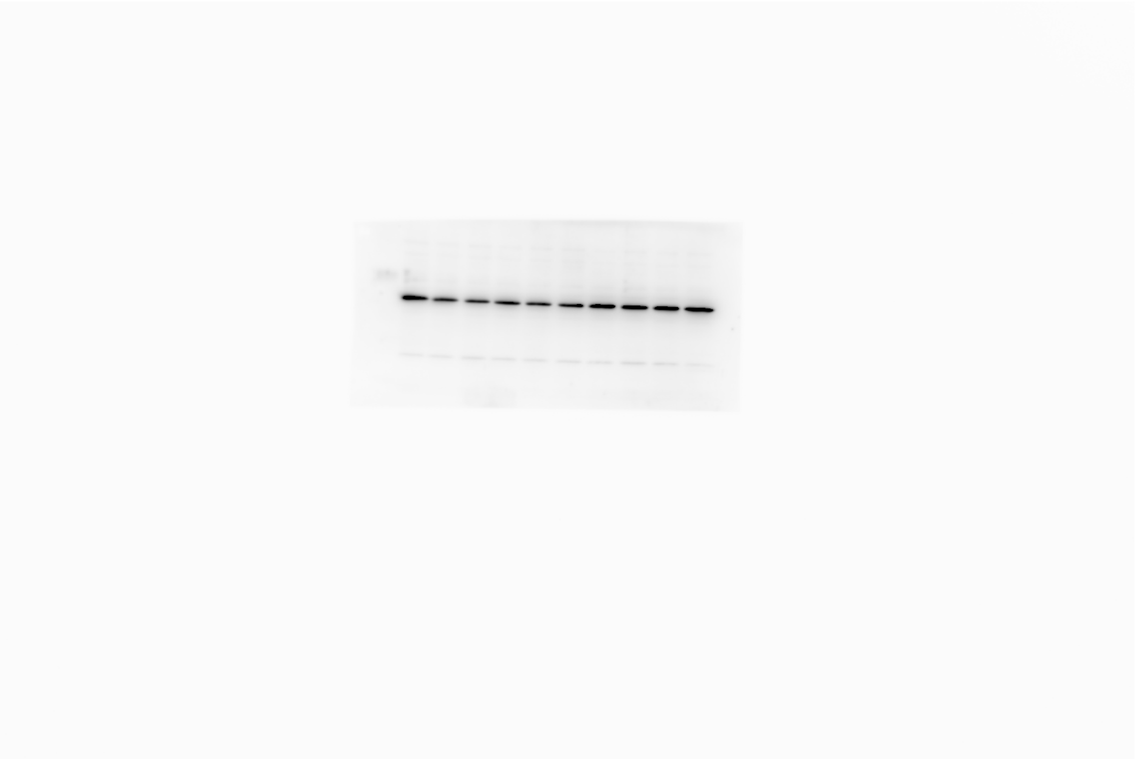

Figure S4

Original image of ACTB.
