# Supplementary material for: Effect of Oxygen Tension Modification During Oocyte Maturation on Porcine Oocyte Quality
Source: Vet Sci. 2025 Oct 3;12(10):954. doi: 10.3390/vetsci12100954 (PMC12568154; doi:10.3390/vetsci12100954)
Supplement: Supplementary file 1 [file vetsci-12-00954-s001.zip › vetsci-3896498-supplementary.pdf]

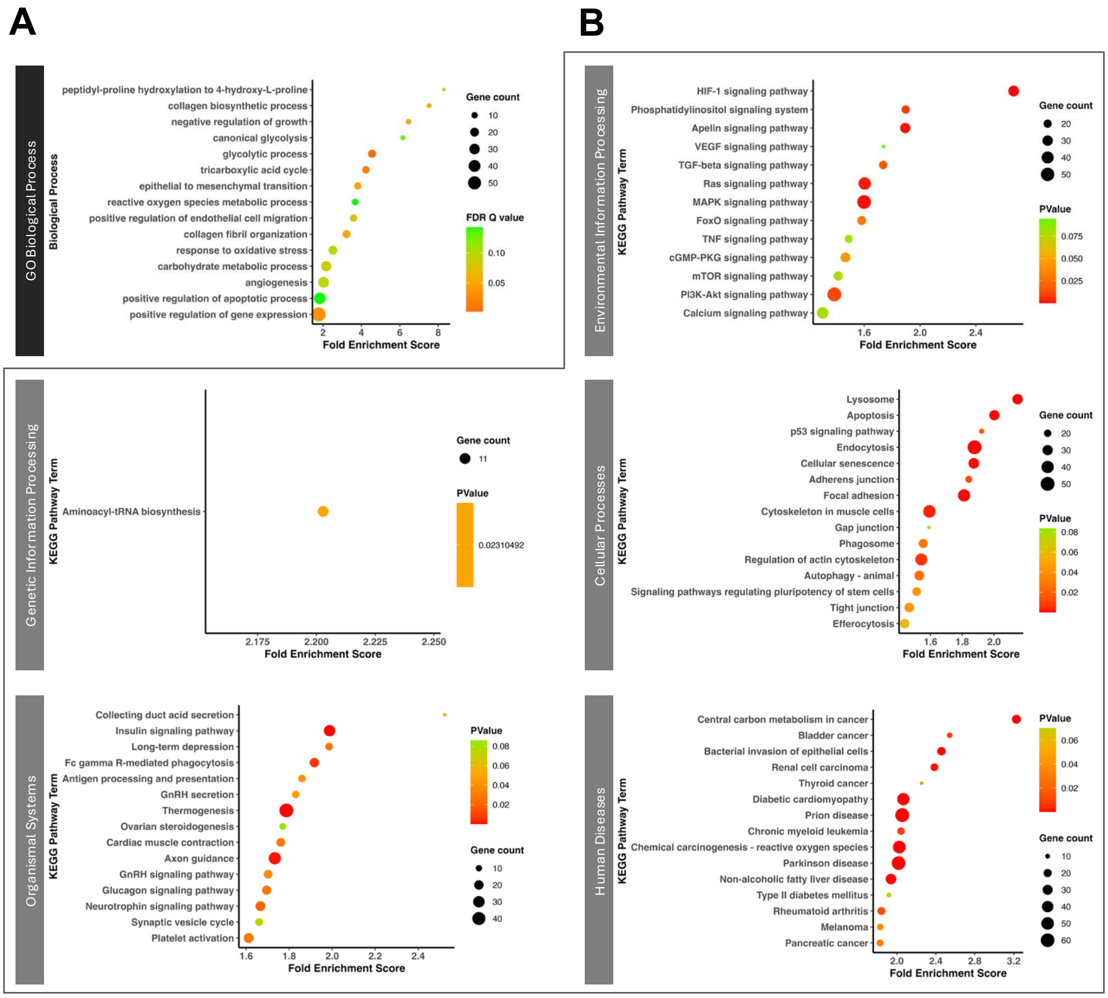

Figure S1

The RNA-seq of the cumulus cells. The top 15 GO terms in Biological process predicted by GO analysis (A). The GO terms were enriched by the differentially expressed genes, as indicated by the fold-enrichment score (colour scale: fold enrichment score; dot size: gene counts). The top 15 KEGG pathways analysis, which subcategories of environmental information processing, Genetic information, Cellular Processes, Organismal Systems, and Human Disease (B). The pathways were

enriched by the differentially expressed genes, as indicated by the fold-enrichment score (colour scale: fold enrichment score; dot size: gene counts).

Table S1. The list of antibodies.

Antibody	Host	Product	Dilution ratio	Catalog_#
TOMM20	Rabbit	MyBioSource	1:2500	MBS820677
GAPDH	Rabbit	proteintech	1:2500	10494-1-ap
ACTB	Rabbit	Cell Signaling	1:5000	4970S
Total OXPHOS Rodent WB Antibody Cocktail	Mouse	Abcam	1:1000	ab110413
Anti-Rabbit IgG H&L (HRP)	Goat	Abcam	1:5000	ab6721
Anti-Mouse IgG H&L (HRP)	Goat	Abcam	1:5000	ab6789
